# Green politics beyond the state: radicalizing the democratic potentials of climate citizens’ assemblies

**DOI:** 10.1007/s10584-023-03550-z

**Published:** 2023-05-30

**Authors:** Mads Ejsing, Adam Veng, Irina Papazu

**Affiliations:** 1 University of Copenhagen, Copenhagen, Denmark; 2 IT University of Copenhagen, Copenhagen, Denmark

**Keywords:** Climate governance, Citizens’ assemblies, Deliberative democracy, Democratic innovation, Mini-publics

## Abstract

In recent years, countries like France, UK, Germany, and Denmark have all carried out climate citizens’ assemblies where a group of representatively selected citizens come together to discuss issues around climate politics and provide policy recommendations to decision-makers. The hope is that these deliberative-democratic innovations can circumvent the flaws of representational politics and help break the existing gridlock around climate politics. In this article, relying on the case of the Danish climate citizens’ assembly that began its work in 2020, we argue that to truly realize the democratic potentials of climate citizens’ assemblies, there is a need to think about how citizens’ assemblies might come to multiply and proliferate in political spaces away from, or at least in addition to, those in and around the state, so they can become local drivers of democratic action and community empowerment. The argument is not that citizens’ assemblies should give up on affecting the state and parliamentary politics altogether, but that we must be careful not to put too much faith in state institutions, and also look for spaces outside the state where the conditions for transformative change and democratic capacity-building currently appear more fecund. Drawing together these arguments, we offer what we call a more radical vision of the democratic potentials of climate citizens’ assemblies, and provide some guidelines for what that would look like in practice.

## Introduction

When I arrive in the Zoom-room, the Danish minister of climate, Dan Jørgensen, is giving his opening speech. He speaks from his living room, and a large green plant is carefully positioned inside the frame. The whole scene conveys a homely feeling. ‘There is a need for a fundamental transformation,’ he says. ‘We are no longer discussing whether climate change is real, but what we are to do about it — and it won’t be easy.’

It is a Saturday morning and more than 100 people have found their way to the virtual room. Most of the participants are members of the new climate assembly, but there are also people from the Board of Technology, the organization in charge of running the assembly meetings. Then there are a few people like me and my colleagues, researchers who are neither members nor facilitators, but are here to take a closer look at this new and exciting democratic experiment: A citizens’ assembly on climate issues.

When the climate minister finishes his opening speech, the frame shifts to Lars Klüver, the director of the Board of Technology. The political context of the climate assembly, Lars explains, is the Danish climate bill, which was adopted in the wake of the parliamentary elections in 2019 after a sudden surge in support for green parties and policies. Moreover, the citizens’ assembly is part of a long democratic tradition of citizen involvement and its design draws on thirty years of experience with consensus conferences and mini-publics. The guiding principle here is inclusive and constructive deliberation.

When Lars is done talking, the participants are divided into groups and sent into breakout rooms to introduce themselves and share their expectations for the climate assembly. Just like that, the first Danish climate citizens’ assembly has begun its work.^1^

This opening story is from the first round of the Danish climate citizens’ assembly (hereafter DCCA), which began with a 2-day online meeting in the last weekend of October 2020. The launch was initially postponed due to the COVID-19 pandemic, but eventually converted into a digital format with members meeting via the online platform Zoom. Over the following months, the assembly met over two full weekends and several evenings to discuss topics around the climate crisis, including transportation, agriculture, technology, financing, and lifestyle (KEFM 2020b). The first round concluded in the spring of 2021, and a second round was initiated in the fall of 2021, which concluded in the spring of 2022 with 73 new recommendations. Among the assembly’s most notable policy recommendations were support for an ambitious production-based carbon tax, the demand that “Denmark should reduce its meat production” (KEFM 2021a, 37), and the position that “it is more important that Denmark reaches its 1.5 degree climate targets … than whether we have economic growth or not” (KEFM 2022, 26; our translation).

Institutional innovations like the DCCA represent interesting democratic experiments in a time where liberal democracies are consistently falling short when dealing with the climate crisis and where scholars in the social sciences have started talking about a crisis, end, or even death of democracy (Levitsky and Ziblatt 2018; Castells 2018; Runciman 2018; Dryzek et al. 2019). In the academic literature on deliberative mini-publics, one of its foremost proponents, John Dryzek, has, together with co-authors, heralded deliberative assemblies as a potential solution to the dual crises of democracy (Dryzek et al. 2019) and climate governance (Dryzek and Niemeyer 2019). Meanwhile, critical voices, such as the democratic theorist Christina Lafont, have pointed out, in her book *Democracy without shortcuts* from 2019, that for deliberative assemblies to become more than “quick fixes” to a broken representative system, and to rise to their true potential, they will have to move beyond the emphasis on formal procedures and consensus-oriented deliberation towards a more participatory perspective that engages assembly participants in processes of collective self-government (Lafont 2019). With the rising interest in climate citizens’ assemblies among both scholars and political practitioners, we believe that a critical discussion of the transformative potentials and limitations of climate citizens’ assemblies is both warranted and particularly well-suited to the interdisciplinary audience of this journal.

In this article, we follow and expand on the critical-constructive position proposed here by Lafont. Using the DCCA as our empirical starting point, we discuss the democratic promises of climate citizens’ assemblies while taking on an multi-disciplinary approach that draws on our own ethnographic fieldwork within the assembly, a digital mapping of the Danish climate movement, as well as recent debates within contemporary democratic theory about the promises of deliberative mini-publics (Setälä and Smith 2018; Thompson and Gutmann 2018; Elstub 2018; Elstub and Escobar 2019; Dryzek and Niemeyer 2019; Dryzek et al. 2019). Relying on these combined insights, we argue that even though climate citizens’ assemblies such as the DCCA are welcome additions to flawed representative liberal democracies, their current democratic shortcomings remain substantial, both in terms of the forms of participation available in these deliberative formats and their ability to foster broader political change. The overarching argument is that in order to realize the democratic potentials of institutional innovations like climate citizens’ assemblies, there is a need to think more about how the democratic energies of these assemblies can come to multiply and proliferate in political spaces away from, or at least in addition to, those in and around the state, so they can become local drivers of democratic action and community empowerment.

In what follows, we begin in Sect. 2 by providing a bit more background about the DCCA, introducing both its specific historical origins and the intellectual tradition of deliberative mini-publics it draws on. Then, in Sect. 3, we situate the DCCA in a broader landscape of climate actors in Denmark, relying on a digital method mapping carried out by the authors in the fall of 2021. Using the map as our starting point, we argue that the DCCA has, by and large, failed to position itself as an important actor within this political landscape, has received limited attention in the general public, and effectuated very little political change. Part of the reason for its relative impotence has to do with a series of institutional shortcomings, most notably its weak political mandate. But it also has to do with the limited role assigned to the DCCA within a broader landscape of civil society actors. Therefore, in Sect. 4, we move on to argue that if the DCCA—and climate citizens’ assemblies more generally—are to become important drivers of a democratic green transition, more work needs to be put into thinking about how citizens’ assemblies can help mobilize more people to take part in the climate agenda, which requires moving beyond state-centric theories of societal change. Following from this argument, we conclude by arguing for a new democratic vision of climate assemblies in which the DCCA is seen as but one of many different engines of public engagement that must work alongside, and in tandem with, other attempts to democratize climate politics today. The fine balance of the argument here is to not put too much faith in citizens’ assemblies like the DCCA as the silver bullet that will solve all of the problems in existing democratic systems, while making sure not to overlook the many and often mundane ways that citizens’ assemblies can be important catalysts of individual and collective change.

## The Danish climate citizens’ assembly: an origin story

In the wake of the national parliamentary elections of 2019, which has been described as the first “green” elections in Denmark, eight out of ten political parties in parliament supported the Danish “Climate Act,” which was formally adopted into law in June 2020. The stated purpose of the Climate Act was to ensure that Denmark will reach its climate targets set out in the Paris Agreement from 2015, which entails reducing greenhouse gas emissions by 70% by 2030 and achieving complete climate neutrality by 2050 (KEFM 2020a). The climate act commits the government to define increasingly ambitious climate targets every 5 years and formalizes the role of an independent expert-led Council on Climate Change that will assist and assess the ongoing realization of Denmark’s climate targets. However, reflecting the broad political coalition behind the agreement, the practical implementation of the bill was to be guided by and balanced against principles such as cost-effectiveness, business development, and national competitiveness (KEFM 2020a, 1).

In the political agreement leading up to the Climate Act, on the bottom of page 3, in just one sentence, it says: “The political parties to the agreement furthermore agree that leading up to the first climate action plan, a citizens’ assembly will be established, where citizens can have their voice heard in the planning of the climate program” (KEFM 2019, 3; our translation). According to people who were part of the political negotiations back in 2019, the line was added late in the process, almost on a whim, to address the concern raised by left-wing parties about the lack of citizen involvement.^2^ That is the history of how Denmark got its first national climate citizens’ assembly. The details of how to actually carry out such an assembly, what it would look like, and what its exact purpose should be were never thoroughly discussed.

In the spring of 2020, the Ministry of Climate, Energy and Utilities (KEFM) was given the task of coming up with a plan for the assembly, which was set to begin its work within the calendar year. To help design and carry out the assembly meetings, and in order to ensure an arm-length distance, the ministry created a public tender that was won by the Danish Board of Technology (DBT). The DBT is a private non-profit company that works with democratic innovations, drawing on an intellectual tradition of deliberative democracy and decades of empirical research into different forms of deliberative assemblies, including the distinctly Danish tradition of consensus conferences (Jensen 2005; Blok 2007). As they write on their website, the format of the citizens’ assembly belongs to a category of “*deliberative* (reflective, dialog-oriented) democratic processes” where a representative group of ordinary citizens are selected and brought together over an extended period of time to discuss and deliberate among themselves about a given topic (DBT n.d.; our translation). According to the DBT, these types of deliberative formats are “excellent at involving citizens in tackling societal challenges that aren’t easy to solve” (Ibid).

A core element of the “citizens’ assembly” format is that its participants are selected by sortition in order to ensure that it is representative of the broader population. In the case of the DCCA, participants were randomly selected with help from the national agency Statistic Denmark, who drew a statistically representative sample of five thousand citizens that were all invited to participate (Statistics Denmark 2020). Among five thousand citizens, 457 (a little less than 10%) accepted the invitation. From those who accepted the invitation, 99 assembly members were selected based on a criterion of maximum representativeness across a number of variables such as age, gender, and geography (Statistics Denmark 2020, 3). The final members were broadly representative of the general citizenry along the aforementioned variables, although skewed towards higher education and higher income, which could have to do with the self-selection bias that comes from people having to be willing to participate in the first place (KEFM 2020c). Although an honorarium of 1000 DKK (app. 130 USD) was offered for each of the two weekend assemblies, a great part of the work undertaken in DCCA took place in their spare time without monetary compensation.

The design that the DBT eventually invented for the first round of the DCCA included two weekend-long open and closing events with a series of around eight shorter and more thematically oriented evening meetings in between. At each meeting, a panel of four to six experts was chosen to give input to the assembly and “ensure quality and balance of expertise” on the respective themes (KEFM 2021a, 8). Originally, the meetings were meant to take place in person, but due to the outbreak of the COVID-19 pandemic in the beginning of 2020, the assembly moved online. Partly as a result, the working method of the assembly shifted to a more writing-oriented method called the OVA format, an abbreviation that comes from the tree Danish words for observation, assessment, and recommendation.

The OVA format was intended to help members carry over insights from one meeting to the next, even with shifting working group compositions, and without being physically present. During assembly meetings, and after having listened to experts, members would split into smaller groups and come up with recommendations for a given theme by first writing down their factual observations based on expert inputs (O), then recording their own evaluations of those inputs (V), before finally committing to a policy recommendation (A). The written product detailing all three stages, usually around a page per recommendation, would then be considered a completed OVA. During a single meeting, working groups would produce several OVAs, and many (but not all) would go on to become part of the final recommendations adopted by the assembly through a collective voting process.

In the late spring of 2021, the Ministry of Climate, Energy and Utilities published the assembly’s first 117 official policy recommendations. The recommendations were, in a number of areas, notably more ambitious than the political line of the government. Nevertheless, the work of the assembly received little public attention, and on the day where a group of selected assembly members were to present their findings to members of the parliament, only politicians from three out of ten parties attended the actual meeting (Tønder et al. 2021). The second round of the DCCA, which began in the fall of 2021, painted a similar picture. This time, the opening and closing weekend assemblies were carried out in person, while evening meetings remained on Zoom. In May 2022, when the assembly presented their second round of 73 recommendations to parliament, history repeated itself: Only a handful of politicians attended the meeting, and the climate minister Dan Jørgensen left half-way through (Ballenstedt 2022).

What explains this lack of interest from the political system? Why are democratically elected politicians not paying much attention to a citizens’ assembly that they have themselves called into action? Why are the recommendations of the DCCA not taken seriously, and what would it take for this to change? These are some of the questions explored in the remainder of this paper.

## Situating the citizens’ assembly in a broader landscape of climate actors

**Fig. 1:**
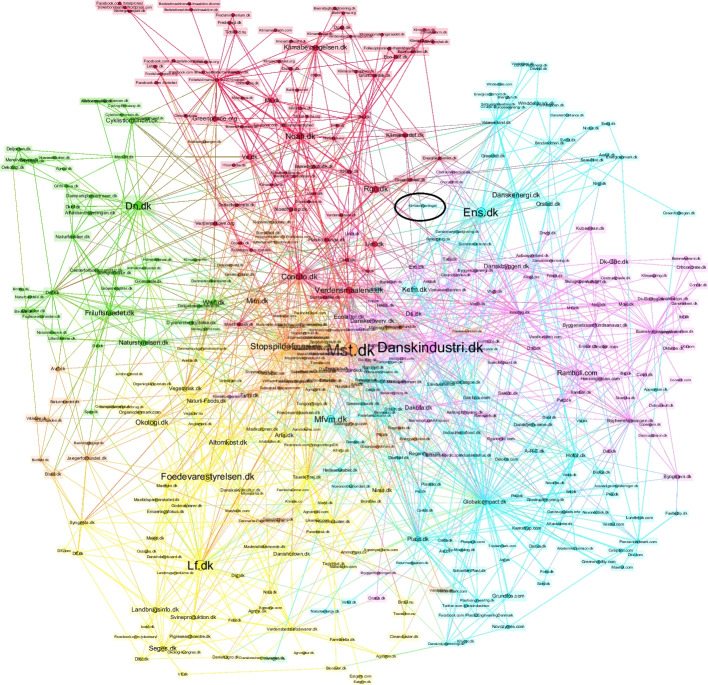
Digital map of the Danish landscape of climate actors. Note: The network is visualized with *Gephi* and spatialized with the algorithm ForceAtlas2 (Jacomy et al. 2014); scaling, 15,000; gravity, 1. With the computation of a community detection algorithm (Blondel et al. 2008), densely interlinked actors (nodes) appear in the colored clusters of the network. For a lengthy discussion of the network and its digital methods, including its theoretical underpinnings, a working paper is forthcoming in the journal *STS encounters.*

The DCCA did not operate in a political vacuum. In the fall of 2020, the authors of this paper carried out a digital mapping of the Danish landscape of climate actors, which is illustrated in Fig. 1. The network map shows how actors—nodes in the network—link to one another through their websites, thus showing what digital methods scholar Richard Rogers calls a “hyperlink-diplomacy” of how actors relate to particular “associational aspirations” (Rogers 2012, 201; see also 2002). While this is not a bullet-proof way of showing actual relationalities between climate actors, it works here as a useful proxy.^3^ The map is made from data harvested with the web crawler tool *Hyphe*, enabling us to import a list of websites (embarking from a list of 33 prominent green NGO’s in Denmark), then receiving a prospect list of the websites they link to, and then iteratively and qualitatively selecting which of these prospected actors to include in the network (see Ooghe-Tabanou et al. (2018) for a thorough description of *Hyphe*). The map includes Danish organizations that have the words “climate,” “environment,” “sustainability,” “nature,” or “green transition” listed on their website landing page. Furthermore, the 16 Danish Ministries and Boards overseeing the climate law are also included. The network entails a total of 472 actors.

Somewhat predictably, the digital map shows that formal public institutions and large business organizations are internally connected and grouped together in one part of the network, to the south-east, while NGOs and civil society groups are located in the opposite end of the network, to the north-west. The different clusters represent, roughly, the energy and infrastructure sector (blue), the construction sector (purple), the agricultural sector (yellow), nature organizations (green), climate NGOs (red), and waste and recycling organizations (orange).

Interestingly, the DCCA, which is circled out in the upper right quadrant, is only part of the map because it is connected to the Ministry of Climate, Energy and Utilities. As a result, it is located in the blue energy and infrastructure cluster, which is otherwise dominated by formal state institutions, private–public companies, and large business organizations. The DCCA’s location on the map reflects the tight connection between the DCCA and the more formal political system, which initiated the assembly.

Perhaps, the most noteworthy element of the map, however, is the sheer number of climate actors within the political landscape of even a small country like Denmark. It is within this vast and complex network of actors that the DCCA has to “make its voice heard,” a task it has largely failed to fulfill.^4^ In contrast, the French climate citizens’ assembly of 2021, which was initiated by Macron in the wake of the yellow vest protests, became center of great public debate in France, even if it eventually failed to deliver on its initial political promises (Steen Nielsen 2020; Dichman 2021). This begs the question of why the DCCA has retained such a marginal role in the Danish public.

### The institutional critiques: lack of political mandate and independence

The standard explanation seems to be the lack of a binding political mandate given to the DCCA (Whyte et al. 2020; Blok et al. 2022). As suggested in the brief origin story told above, the line about the citizens’ assembly was added to the agreement about Danish climate bill last minute, and there was never any clear or explicit political mandate given to the assembly. As a result, the political purpose of the DCCA was never spelled out in detail, and neither were the procedures meant to follow up on the work carried out by the assembly itself. In other words, two of the most vital elements of “best practice” when it comes to designing a citizens’ assembly, namely defining its purpose and creating transparent follow-up procedures (OECD 2020, 9), were entirely overlooked.

**Fig. 2:**
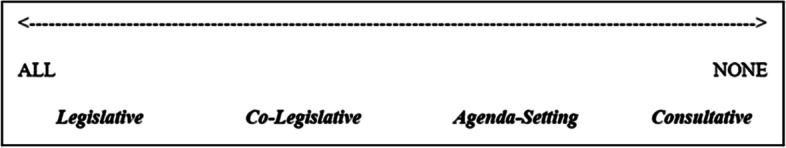
The different mandates of citizens’ assemblies. Note: From Mulvad and Ask Popp-Madsen 2021

In practice, therefore, the DCCA has by and large taken on a what we might call a “consultative role” with no formal agenda-setting or decision-making power. According to Mulvad and Popp-Madsen (2021), we can distinguish between four different types of political mandate given to citizens’ assemblies, ranging from a purely *consultative* mandate, like in the Danish case, to an outright *legislative* mandate where the assembly is given full legislative competence over a given policy area, in this case climate policies (Fig. 2). In between those two extremes are an *agenda-setting* mandate, where the citizens’ assembly “is endowed with the power to actually frame the subsequent legislative handling of a given matter,” and a more demanding *co-legislative* mandate, where decision-making power is shared between an elected parliament and a citizens’ assembly in a sort of bi-cameral approach (Mulvad and Popp-Madsen 2021, 81–82).

The purely consultative role of the DCCA has given rise to critiques from both political experts, civil society organizations, and green social movements, who have suggested that the DCCA is little more than an extended “focus group,” and that there is no incentive for politicians to actually care about the recommendations produced by the DCCA (Whyte et al. 2020). One current member of parliament even described the DCCA as a “a symbolic faux politics” (Scavenius 2020). Thus, if the DCCA is to be taken seriously as a democratic innovation, the critics argue, its political mandate must be strengthened. The independent Danish Climate and Transition Council (KOR) has suggested, for example, that the DCCA take on a political “agenda-setting” role that commits the parliament to follow up on the recommendations provided by the assembly in a number of legally codified ways, for example, by voting on its recommendations in parliament (KOR 2020, 8).

Closely related to the critique of a lacking political mandate is a range of other institutional critiques, which can also help explain why the DCCA has not had the impact its proponents had hoped. From the very beginning, the process of establishing and designing the assembly has been closely tied to the formal political system in the form of the Ministry of Climate, which breaks with the principle of arm’s length distance meant to ensure deliberative assemblies a certain degree of independence from the political system. Moreover, resources dedicated to developing and carrying out the DCCA were extremely sparse. To compare, the French climate citizens’ assembly mentioned above had its own independent secretariat and a budget of 5.4 million euro, whereas the Danish one had only a fraction of that.^5^ Combined with the lack of political mandate, these factors—the lack of an independent secretariat and the low budgets—prevented the DCCA from taking on an independent role and becoming a public actor in its own right.

### The process-oriented critiques: political constraints and digital format

The shortcomings of the DCCA as a democratic innovation go deeper than the institutional critiques of a lacking political mandate and organizational independence suggest. While these critiques are generally are well-founded, they risk missing the bigger picture. There is no doubt that a stronger political mandate could help alleviate the sense of irrelevance and the lack of public attention around the DCCA, but there remain several reasons why even a citizens’ assembly with a stronger political mandate, and an independent secretariat, might not deliver on its promises. First of all, the work undertaken by the DCCA was, from the very start, straightjacketed by the political constraints set out by the existing political system. As shown in the opening scene, its members were told to adhere to the principles laid down in the Danish Climate Act and that the assembly’s recommendations must take into account “sustainable business development and Danish competitiveness, sound public finances and employment, and that Danish business must be developed rather than diminished” (KEFM 2020a, 1). This political framing limits the range of possibilities and imaginaries available to the assembly participants when it comes to climate-related topics like agriculture, carbon tax, and consumption patterns.

Adhering to these principles couches the work of the DCCA in a type of “ecological modernization” where responding to environmental and climate-related problems is only feasible, sometimes only imaginable, if doing so simultaneously stimulates economic production and perpetual growth (Hajer 1995),^6^ a combination that is beginning to look more and more like a techno-modernist pipe dream today (Parrique et al. 2019; Hickel and Kallis 2020; D’Alessandro et al. 2020). On the more positive side, the normative force of these principles was never absolute within the work of the assembly. As mentioned already, the members of the DCCA came out of the second round stating that it is more important for Denmark to reach its climate targets than to sustain its economic growth, which flies in the face of the economy-oriented guiding principles of the Climate Act (KEFM 2022a, 26). This suggests, at the least, that citizens’ assemblies might not be so easily governed by “external constraints,” and that if given the chance, assembly members are willing to take on, sometimes even break out of, the constraints under which they have arisen. That being said, the degrowth recommendation (if we can go so far as to call it that) received notable pushback from the establishment. The central administration, in its formal responses to the assembly’s policy recommendations, dryly responded that “the government has not addressed the question of economic growth. Economic growth is not considered to be a hindrance to reaching the climate targets.” (KEFM 2022b, 6; our translation).

Another constraining feature of the DCCA has less to do with the external constraints and more with the inner dynamics of the assembly itself. Some of these dynamics are conditional upon the specific design developed for the DCCA, which was in part necessitated by the arrival of the global pandemic. Others, however, cut much deeper and might be considered an integral part of the very format of these deliberative assemblies themselves. In the first category is the turn to the format of the OVAs described above, which became implemented in response to the assemblies moving to the online platform Zoom, as a tool to preserve insights from one session to the other. The upshot was that the work of the DCCA became centered around an object of writing, which to some members of the assembly felt overly academic. Several participants expressed uncertainty about both the OVA concept itself—such as how to distinguish between observation, evaluation, and recommendation—and the difficulties of having to put their thoughts into writing, while others, often participants with longer educational backgrounds, seemed to thrive in this format. In other words, the turn to OVAs contributed to a potential schewing of the voices that were able to make themselves heard in the process, and even more so the voices that made it into the final written recommendations.

### The structural critiques: representational logic and limited participation

In a more fundamental way, however, some of the democratic potentials of the DCCA were circumscribed from the very beginning by the representational logics underpinning the deliberative format of citizens’ assemblies themselves. As the organizers from the DBT repeatedly made clear, the format of citizens’ assemblies follows a representational logic where participants are meant to channel or mediate the opinions of the broader public under informed conditions. Ideally, the final recommendations of the DCCA should reflect the opinions and perspectives of the general population under better informed conditions. The participants are not, therefore, conceived of as agents of change themselves, but rather as surrogates who transmit and make available the informed opinion of the populace, while the political power remains with the existing system. As the DBT writes on their website, “these methods respect representative democracy by leaving the final decisions to elected politicians” (DBT n.d.). One of the concerns, from a democratic standpoint, is that this limited way of understanding, and involving, citizens in political decision-making processes enacts an overly restricted notion of citizen participation.

There are several examples from the assembly meetings in the DCCA where this limited deliberative logic of representation came up against what we could call a more empowered, radical notion of assembly participants as active and self-governing democratic citizens. From the very first meeting of the assembly, where a group of members wanted to create their own Facebook group to discuss the themes of the assembly outside the structures laid down by the DBT (this suggestion was immediately closed down by the organizers who feared losing control with the process), to the critiques and frustrations aired in public by participants in the wake of the two first assembly rounds. This suggests that even if the format of deliberative assemblies casts citizens in the role of mediating agents, actively involving citizens in democratic processes inevitably entails a measure of both unpredictability and ungovernability, where *particular* citizens, with their own desires and powers, can refuse to be shoehorned into the limited role assigned to them. In this recognition lies, we want to argue, an incipient resource for a more radical democratic understanding of the citizens’ assemblies, which we spell out in more detail below.

Even taken together, all of these critiques—the lack of political mandate, the external and internal constraints, and the limitations of the format itself—do not amount to a complete refusal of the political relevance and democratic promise of climate citizens’ assemblies like the DCCA. What we are suggesting, here, is that the standard critiques about a lacking political mandate must be supplemented and expanded with critiques about the democratic logics and inner workings of the DCCA itself, and the recognition that there might be something about the nature of deliberative citizens’ assemblies that prevent them from becoming the solution to the ongoing ecological and democratic crises that some people seem to hope. Part of the problem with focusing too narrowly on the mandate critique is that it simultaneously puts both “too much” and “too little” faith in the democratic potentials of citizens’ assemblies.

*Too much* faith, because it suggests that if only the DCCA had a stronger mandate, it would necessarily bring about real and transformative change. While there is no doubt that a climate assembly with a stronger mandate could shift, and possibly in quite radical ways, the power balances of existing parliamentary politics, a national climate assembly would still, from the perspective of ordinary citizens, remain a distant representational institution that only actively engages a small part of the population. Moreover, it would continue to be limited by the dynamics that are perceived to be necessary for it to be able to speak to the formal political system, such as the need to speak to and follow the rhythms of formalized legislative processes (Lafont 2019; Mulvad and Popp-Madsen 2021). In other words, a stronger mandate would certainly make a difference—one that is worth fighting for—but it is not the entire story.

On the other hand, the mandate critique might put *too little* faith in citizens’ assemblies, because it focuses too narrowly on the relationship between the DCCA and the formal political system, while the real democratic promise of climate citizens’ assemblies might lie not (only) in the ability to affect a flawed representational political system, but (also) in the ability to promote and extend spaces for citizen involvement and democratization beyond existing political institutions. For the DCCA to realize its potential as a driver of a radical democratization of climate politics, it must be conceived not only (or even primarily) as a representational mechanism that brings perspectives from ordinary citizens to political leaders in an attempt to affect state politics, but rather as a vehicle of societal mobilization at large. To return to the map that we opened this section with, the democratic connections of the DCCA, its associational aspirations, must extend more forcefully in the direction of the broader civil society represented by the red cluster in map. Therefore, in the next section, we turn to look more closely at how one might begin to think of the democratic potentials of climate assemblies in this other way.

## Realizing the radical democratic potentials of climate citizens’ assemblies: routes towards increased political influence

The democratic promises of citizens’ assemblies lie not only in their ability to meaningfully include citizens in processes of political decision-making, but also in their ability to foster societal change and produce new solutions around a given topic, such as climate change (Dryzek and Niemeyer 2019; Willis et al. 2022). However, the recent empirical examples of climate assemblies in places like Denmark, France, and elsewhere have shown limited success in their ability to foster any transformative political change, when it comes to instituting concrete and ambitious climate policies. In the previous section, we presented some of the underlying institutional and philosophical reasons why that might be the case. In this section, we want to do something that is less common within the discipline of democratic theory, namely move beyond the familiar stances of denouncement and critique, and instead try to identify, even prescribe, new routes of action that might help bring climate citizens’ assemblies—and our academic thinking about them—out of the current deadlock.

Following loosely the types of critiques identified above—the lack of mandate, weak institutional setup, its internal dynamics, and the limited representational logic—we can already identify at least four corresponding routes towards increased political influence. These are summarized in Table 1.

**Table 1:** Strategic routes of increased influence for climate citizens’ assemblies

	Strategy	Influence mechanism	Desired outcome
(1)	Mandate	Vertical link—between assembly and formal political system	Affecting the formal political system of state politics through policy change and public debate
(2)	Institutional setup		
(3)	Participatory transformations	Horizontal links—between assembly and civil society	Mobilizing citizens and building collective power outside, or in addition to, formal political spaces
(4)	Local and regional assemblies		

The *first* and most obvious route to increased influence would be providing climate citizens’ assemblies with a stronger and legally binding political mandate. By ensuring that the final recommendations of the assemblies will be turned into law, either through direct adoption or some form of co-legislative process, the assemblies would by definition be set up for making change. Moreover, in addition to the direct influence on policy-making, a stronger mandate could help create a more engaged and sustained public conversation around the assemblies with both media and citizens having a material interest in understanding the work carried out within an assembly that has been ascribed formal legislative power. For these reasons, there is no doubt that pushing for a stronger political mandate will have to be *part of* the ongoing struggle to increase the political influence of climate citizens’ assemblies.

There are, however, several problems of varying gravity with the strategy of focusing only on the mandate: the first being that even if climate citizens’ assemblies were to be given a stronger political mandate, it remains unclear how much of a difference that can actually make. As mentioned above, the work that goes on in the assemblies is constrained, for better or worse, by the external political context and the internal dynamics and designs of the deliberation that takes place within the assemblies. In the end, the recommendations made by the participants of the assemblies must fit into an existing juridical and political system to which they must seem appropriate, which puts at least a limit to the kinds of transformations that can be expected from these types of assemblies. Yes, the DCCA did come out with policy recommendations that were more radical than the current parliamentary status quo, especially around themes like agriculture and economic growth where vested interests often hold a sway over parliamentary positions. But not only were these recommendations not taken up by the political establishment, they also remain relatively marginal advancements in the broader context of the ecological and climatic emergency. That does not render them useless or unimportant, but it does highlight the need to think about the transformative potentials of climate assemblies beyond the mandate question.

Therefore, the push for a stronger political mandate must, at least, be coupled with a *second* strategy, namely the provision of a more independent and economically viable institutional setup for the work of the assembly. This includes ensuring that the climate citizens’ assemblies have their own budgets and secretariats, which can help ensure their independence from the existing political structure and incumbent interests. This is important not only for the work that goes on within the assembly, but also for the opportunity for the assembly to take on an independent role in the public sphere. In the case of the DCCA, in part because the secretary functions were carried out by civil servants in the Ministry of Climate, who could not speak publicly on behalf of the assembly, the DCCA never gained a collective and independent voice in the public debate. Instead, members of the assembly were left with the opportunity to speak with the media only on their own behalf, as citizens rather than as spokespersons for the assembly as such. This can be contrasted, again, with the French case where the assembly came out and publicly criticized Macron for the lack of action taken on their recommendations, rating him a somber 3.3 on a scale of 0 to 10 (Citoyenne Pour le Climat 2021, 161). Even if the French assembly did not manage to implement legislative change, they influenced public opinion and helped ensure the existence of a public conversation about the work of the assembly, something that never materialized in the Danish case, except for a few opinion pieces in national newspapers.

There is an even deeper problem, however, with focusing on mandate and institutional setup as routes towards increasing the influence of climate citizens’ assemblies. The existing parliamentary politicians, who have to provide the binding mandate, are exactly the ones who stand to lose the most from doing so: their own political influence. In other words, part of the problem that climate citizens’ assemblies are invented to address—the insufficiencies of existing representative parliamentary institutions—is also part of what hinders assemblies from rising to influence in the first place. Despite calls for stronger mandates from academic experts and climate activists, there is no reason to believe that the situation will change anytime soon. In fact, in the case of the DCCA, it is much more likely that the work of the assembly will be terminated now, after the second round, than it would be ascribed more power. Part of the strategic question therefore is what do we do in the meantime? Are there other routes of influence that do not depend on this (broken) link between the assembly and the formal spaces of political and legislative power?

This leads us to at least two alternative ways of thinking about the influence and relevance of climate citizens’ assemblies that are sometimes overlooked, or at least downplayed, not only in the public debates about climate assemblies, but also in the literature on deliberative assemblies. These strategies have to do with what we might call the more horizontal and radical (as opposed to vertical and incremental) dimensions of the democratic potentials of citizens’ assemblies.

The first of these, and the *third* strategic route of influence, is tied to the transformational experience of being part of a citizen’s assembly. Listening to the members of these assemblies, many will tell you that taking part in these democratic processes has been a transformative experience, which has affected not only their attitudes and knowledge about climate politics, but also their communities outside the assemblies where they have engaged in new conversations with their fellow citizens. In the Danish case, more than three out of four of the members of the DCCA expressed that the experience had made them more likely to participate in other civil society initiatives around climate change (Bukhave and Friis 2021, 10). These types of individual-level effects are often treated as insubstantial, or at least secondary, benefits of climate citizens’ assemblies. In the public debate, what takes center stage is the extent to which these assemblies can affect the parliamentary and legislative status quo. But what if that is not the only, or even the primary, measure of the climate assemblies’ success? Contrary to deliberative notions of the assembly participants as randomly selected and interchangeable representatives of a broader population, the actual and embodied members of the assembly do not live and operate in a vacuum; they are full and whole beings with the potential to affect their communities outside of the assembly. In other words, citizens transformed through their participation in climate assemblies have the potential to produce ripple effects that reach far beyond the work of the assemblies themselves.

When we talk about a single national assembly with less than a hundred citizens, these transformative effects are, of course, miniscule in the larger scheme of things. But this is exactly what brings us to the *fourth* potential route of influence, which might just be the single most important way of expanding and radicalizing the democratic promise of climate citizens’ assemblies, that is, multiplying and proliferating the existence of citizens’ assemblies across several scales and sites of the political landscape. If the very experience of taking part in one of these assemblies, and the democratic education and empowerment involved in that participation, truly makes up their most transformative aspects, is that not an argument for expanding and distributing these assemblies more broadly? By expanding the existence of climate citizens’ assemblies to both regional and local levels, they might become actual democratic engines of change, which can help mobilize and engage citizens in a green transition (Lafont 2019).

While there are no guarantees that local or regional assemblies will be more effective at tackling climate change than national ones, there are reasons to be optimistic.^7^ Firstly, local assemblies and its participants are closer to the affected communities where climate change is felt and where new policies have to be made, which helps ensure local engagement and ownership over political decisions. Secondly, community-level assemblies are often less constrained by formal party politics, both because they can be initiated bottom up by civil society actors to help ensure political independence (although with the related risk of lack of integration into the existing political system) and because they do not have to fit neatly into the often highly structured and ritualized temporal rhythms of state politics. Finally, and most important for our argument here, even in cases where local assemblies do not turn out to perform better at the level of immediate policy implementation, they remain vehicles of mobilization that interpellate citizens into becoming transformative actors of change in their communities around climate-related issues.

The skeptical reader might object here: How is this call for multiplying and radicalizing existing climate citizen’s assemblies different from calling for a stronger mandate that is unlikely to ever materialize? Is the idea of myriad climate citizens’ assemblies spreading across the political landscape not equally, or more, naïve? The short answer here is no, not necessarily, because the change is already happening. In contrast to a national assembly, citizens’ assemblies at other levels need not be initiated by formal state powers, but can exist at many different levels of political influence and formality. In recent years, and with increasing speed, climate assemblies are being taken up by everything from municipalities, that want to know how to properly involve their citizens in local issues around environmental matters, to green social movements that use the radical democratic aspirations of climate assemblies as a rallying point.

Paradoxically, these latter movements, such as Extinction Rebellion, sometimes fall into the trap of understanding the democratic potentials of climate assemblies in a too narrow way, suggesting that a citizen’s assembly on climate and ecological justice could “break the deadlock” and “put fairness and justice at the center of decision-making” if only it had a stronger mandate (Extinction Rebellion UK n.d.). As we have argued, however, the real democratic potentials of climate citizens’ assemblies lie not so much in any single assembly coming up with climate “solutions,” as they lie in the ability of many assemblies, working in many ways and, at many levels at once, mobilizing more people into the broader political struggle for a more sustainable future. A single national climate citizens’ assembly, even one with a directly binding mandate, would not provide any quick fixes. Considering the scale and intensity of the challenges facing the world today, they are at best marginal improvements of a deeply problematic status quo.

Focusing on what we have called the third and fourth routes of democratic influence of citizens’ assemblies might help expand the political focus away from the relationship between citizens’ assemblies and the formal politics state and towards the many and often complex links between DCCA and civil society. The hope is that this shift in focus might help avoid channeling too much of available democratic energies into flawed systems of representational politics, which are notoriously slow to change. This risk is constantly present, as we have seen in cases such as Syriza in Greece or Podemos in Spain, where originally vibrant and popular democratic mobilizations lost part of their broader momentum when the movements focused their energies on reforming the politics of hierarchical national parliaments and international organizations like the EU. Not because the work of reforming these formal institutions should not be taken on by democratic movements, but because that is only one route towards potential influence and change.

This way of approaching citizens’ assemblies amounts to a notably different way of understanding the democratic potentials of climate assemblies than the ones proposed by deliberative theory. Against the deliberative model of citizens’ assemblies, we offer here what we call a “radical” vision, which echoes broader theoretical traditions of radical, agonist, and participatory democratic theory that seek to expand democratic spaces to more realms of our current life than formal state politics, including for example our workplaces and local communities. Thus, a radical approach to climate assemblies would be one that envisions the assemblies as vehicles of greater democratizing of climate politics in places and at scales where it has hitherto been absent. It is important noting here that this radical vision of climate assemblies is not a wholesale rejection of the theoretical work that already exist around deliberative mini-publics, but an attempt to build on and extend related contributions, such as Dryzek and deliberative democrats’ call for a system-oriented approach to deliberative assemblies that focuses on multi-sited and multi-scalar distribution (2016), and Lafont’s more agonistic and participatory theory of assemblies as vehicles of self-government (2019). An idealized conceptual overview of the two visions is summarized in Table 2.

**Table 2:** Two alternate democratic visions of climate citizens’ assemblies

	Democratic logic	Participatory ideal	Political aim	Mechanism of change
Deliberative	Representation	Mediation	Legitimacy	State politics
Radical	Empowerment	Transformation	Mobilization	Multi-sited democratic action

We want to emphasize here that these two visions are not, neither in theory nor in practice, mutually exclusive or necessarily in competition. They are a matter of both-and. In order to increase the political influence and relevance of climate citizens’ assemblies, there will have to be people working to push for a stronger link between the assemblies and the formal political system, through more binding forms of legal-political mandates and more robust institutional setups. Meanwhile, others will have to push for and help facilitate the spread of inclusive assembly processes outside formal spaces of (state) power that help mobilize and build momentum around the climate crisis through the fostering of horizontal relations. This is also why we are not suggesting that the mandate critique should be put to rest. We do believe, however, that the arsenal of critiques of the impotency of climate citizens’ assemblies must be expanded and supplemented with alternative strategies and routes for transformative change along all the avenues of influence identified above (Table 1). As suggested by political theorist Connolly and his concept of “a politics of swarming,” what we need today, considering the many and complex crises unfolding around us, are many different kinds of actions at multiple sites and multiple scales all at the same time. We need to work on several fronts at once without losing sight of the hope that these dispersed and multi-sited efforts might come to resonate and work together to foster change at a greater scale (Connolly 2013, 2017). That might seem like a radical hope, but it might very well be the best shot we have.

## Concluding remarks: pluralizing democratic engagements in a green transition

On the face of it, climate assemblies like the DCCA seem to offer promising democratic solutions to the ongoing crises of existing liberal democracies. However, for reasons laid out in this article, both institutional and otherwise, they often come to be viewed as public and political “failures”—but what is democracy if not a process of perpetual failure and learning? Even when climate assemblies fail, they often bring important topics to the forefront of public debate and, even more so, affect the people who partake in these assemblies in transformative ways. For these reasons, climate citizens’ assemblies, despite their many flaws, should be seen as a welcome innovation in a time when representative liberal democracies seem to be losing their political efficacy and their grip on the political imaginaries of their citizens.

What we have wanted to do in this article is offer a new and more “radical” perspective on the democratic potentials of climate assemblies that tends to be overlooked, or at least downplayed in deliberative theories of citizen participation, as well as in the public responses to the Danish climate citizens’ assembly, namely, that the success of climate assemblies should not only be judged on their ability to affect the formal political system. They should also, and perhaps even more so, be judged on their ability to include and mobilize ever broader parts of the population into the fight for a more sustainable world. In order to realize these potentials, to realize the radical democratic aspirations of climate assemblies, we have to start thinking of them not so much as turned inwards towards parliaments and state administrations, but (also) as vehicles of change and mobilization that can, and must, spread out across democratic societies and into local neighborhoods, where they can help build new, empowering, and inclusive democratic processes—alongside the many other efforts, institutional or otherwise, that already exist and seek to build collective power and begin new processes of democratic change.

A promising democratic theory and strategy around climate citizens’ will have to focus not only on the “internal” links between the assemblies and formal state politics, but also on the “external” links between the assemblies and the broader landscape of civil society and ordinary citizens it is able to influence and mobilize. Moreover, climate assemblies should not be seen as a silver bullet that can solve the dual crisis of climate and democracy, if only we get their designs right. Even in the most optimistic light, climate assemblies must be viewed as one effort among many others that will have to take place at other levels and by other actors in other sites and that hopefully all can come to work together, resonate, and bring about transformative change at larger scales.

Climate citizens’ assemblies, like the DCCA, do not operate in a political vacuum, and external political conditions often make it difficult for them to make an immediate impact on sedimented parliamentary and state-led politics. But it is exactly because they do not operate in a vacuum, but are—or can be—connected to a myriad of other people, places, and spaces of power, that we have reason to remain optimistic about their democratic potentials, not as a simple or quick fix-all solution, but as one more wave in an ocean of slow, grinding, and ever-ongoing democratic struggles for a livable future on a rapidly warming planet.

## Data Availability

Not applicable.

## Footnotes

1 The reconstruction of the scene relies on field notes from the first day of the Danish climate citizens’ assembly, which took place on the 24th of October 2020.

2 Personal communication on the 26th of October 2021 with a member of the left-green party, Alternativet, who is usually credited for getting the line about the citizens’ assembly into the political agreement.

3 To investigate the practical relevance of the digital mapping, it was presented to and discussed with members of the network through a workshop carried out in the spring of 2022, which emphasized while this mapping is neither the only or necessarily right way to illustrate a complex field, it is helpful as a heuristic advice. For a more thorough discussion on the considerations about the digital mapping, see our forthcoming paper in the journal *STS encounters*.

4 While the map itself does not say anything about the amount of public debate in Denmark, a simple search in national Danish newspapers reveals that between 2020 and 2023, a total of 62 articles included the word *klimaborgerting* (“climate assembly”), and much fewer had the DCCA as their main topic.

5 No official budget is publicly available, but people involved in the process suggest that the number might have been as low as 200,000 euro (Mosbech, 2021).

6 See also *An Ecomodernist Manifesto* (The Breakthrough Institute 2015) for a defense of this kind of thinking.

7 We want to briefly note here the potential concern that these local assemblies might be more prone to make short-sighted decisions for the benefit of the local community in the vein of phenomena such as “Not In My Backyard” (NIMBY) problems in relation to new energy projects and the like. While this is a risk, we see no reason to believe that this concern would be exacerbated in local climate citizens’ assemblies vis-a-vis existing regional or municipal representational politics that are also shaped by local interests. If anything, our fieldwork in the DCCA has reminded us how willing and capable “ordinary” citizens are to engage with each other in meaningful ways, even around complex and difficult issues such as climate politics.
